# Ozboneviz: an Australian precedent in FAIR 3D imagery and extended biodiversity collections

**DOI:** 10.1093/biosci/biaf064

**Published:** 2025-06-10

**Authors:** Vera Weisbecker, Diana Fusco, Sandy Ingleby, Ariana B J Lambrides, Tiina Manne, Keith Maguire, Sue O'Connor, Thomas J Peachey, Sofia C Samper Carro, David Stemmer, Jorgo Ristevski, Jacob D van Zoelen, Pietro Viacava, Adam M Yates, Erin Mein

**Affiliations:** College of Science and Engineering, Flinders University and the Australian Research Council Centre of Excellence for Australian Biodiversity and Heritage (CABAH), Adelaide, South Australia, Australia; College of Science and Engineering, Flinders University and CABAH, Adelaide, South Australia, Australia; Mammal collection, Australian Museum, Sydney, New South Wales, Australia; Society and Education, James Cook University, Cairns, Queensland, Australia; School of Social Science, University of Queensland, Brisbane, Queensland, Australia; Natural Science Collections, South Australian Museum, Adelaide, South Australia, Australia; College of Asia and the Pacific, Australian National University and CABAH, Canberra, Australian Capital Territory, Australia; 3D Digitisation, Australian Museum, Sydney, New South Wales, Australia; College of Asia and the Pacific, Australian National University and CABAH, Canberra, Australian Capital Territory, Australia; Collection Manager, South Australian Museum, Adelaide, South Australia, Australia; Max Planck Institute of Geoanthropology in Jena, Germany and Griffith University in Brisbane, Queensland, Australia; College of Science and Engineering, Flinders University and CABAH, Adelaide, South Australia, Australia; Australian National Wildlife Collection, Commonwealth Scientific and Industrial Research Organisation, Canberra, Australian Capital Territory, Australia; Earth Sciences at the Museum and Art Gallery of the Northern Territory, Alice Springs, Northern Territory, Australia; College of Science and Engineering, Flinders University and the Australian Research Council Centre of Excellence for Australian Biodiversity and Heritage (CABAH), Adelaide, South Australia, Australia

**Keywords:** paleontology, zoology, archaeology, museums, morphology

## Abstract

Billions of specimens are in biodiversity collections worldwide, and this infrastructure is crucial for research on Earth's natural history. Three-dimensional (3D) imagery of specimens is an increasingly important part of the digital extended specimen network of metadata. Open-access, high-fidelity 3D imagery of biodiversity specimens improves researcher efficiency and equity and increases public engagement with collections. We introduce Ozboneviz, an open-access collection of FAIR (findable, accessible, interoperable, reusable) 3D imagery aiming to enhance research capacity in Australasian vertebrate skeletal morphology. Ozboneviz is an Australian test case demonstrating the feasibility of creating multi-institutional, FAIR 3D biodiversity imagery collections. We outline its project design, challenges, and use by the international research community. We then discuss the urgent need for investment in infrastructure and curatorial support to progress the digitization of Australian biodiversity collections in a way that maximizes stakeholder benefit and facilitates 3D data discoverability and retrieval.

Biodiversity collections have been the mainstay of research in areas such as evolutionary biology, paleontology, archaeology, taxonomy, and ecology (Holmes et al. 2016, McLean et al. 2016, Meineke et al. 2018, Lyman 2019, Hilton et al. 2021, Beck et al. 2022). They also have critical industrial applications such as for pest and invasive species management, biosecurity, agriculture, and conservation, as well as playing a major role in public and tertiary education (Suarez and Tsutsui 2004, Lyal et al. 2008, Cook et al. 2014, Ball-Damerow et al. 2019, National Academies of Sciences and Medicine 2020, Atlas of Living Australia et al. 2023). Accessing the three-dimensional (3D) morphology of biodiversity specimens is therefore a core requirement across the academic, commercial, and government sectors. Although curation of physical vouchered specimens remains crucial, high-quality 3D imagery can facilitate education and research programs simultaneously and efficiently.

For many stakeholders, access to physical biodiversity collections can be inefficient, costly when travel to collections is required, and potentially damaging to specimens through repeated handling (Page et al. 2015, Davies et al. 2017, Hipsley and Sherratt 2019, Johnson et al. 2023). Public engagement with these mostly taxpayer-funded research collections is also limited to curated exhibition initiatives, principally in museums (Powers et al. 2014, Boyer et al. 2016, Nelson and Ellis 2019, Blackburn et al. 2024). Increased access to these collections can therefore improve research efficiency, reduce inequities in the research community, and deepen and diversify public engagement in this important infrastructure (Cook et al. 2014, Drew et al. 2017, Hipsley and Sherratt 2019, Nelson and Ellis 2019, Hedrick et al. 2020, Lendemer et al. 2020, Atlas of Living Australia et al. 2023, Johnson et al. 2023, Blackburn et al. 2024).

More equitable specimen access can be, in part, achieved through high-fidelity 3D imagery that acts as a digital avatar of a physical specimen (Hipsley and Sherratt 2019). An ongoing revolution in 3D digital imaging has made the creation of these avatars increasingly affordable (Rowe and Frank 2011, Boyer et al. 2016, Davies et al. 2017, Nelson and Ellis 2019, Weisbecker et al. 2024). Photogrammetry, structured light, and laser scanning can be particularly inexpensive. However, costlier modalities such as magnetic resonance imaging or X-ray computed tomography (CT) are also routinely used and are often more informative than the physical examination of a specimen because they can reveal internal or small-scale features that are difficult to observe with the naked eye or without destructive sampling (Hilton et al. 2021, Kimura 2023, Blackburn et al. 2024). 3D imagery can be used to retain information prior to destructive sampling or insure against total data loss during disasters (NSWDC 2007, Escobar 2018, Tyler et al. 2023). It can be easily and quickly disseminated (Boyer et al. 2016, Davies et al. 2017, Hipsley and Sherratt 2019, Blackburn et al. 2024). When curated well, 3D imagery can also be an important part of the digital extended specimen (Webster 2017, Lendemer et al. 2020, Hardisty et al. 2022), the connected web of data and metadata associated with a single specimen. Use of 3D imagery is rapidly increasing in biodiversity research (Čerňanský and Syromyatnikova 2019, Early et al. 2020, Dong et al. 2022, Maden et al. 2023), outreach, and education (Ulguim 2018, Flemming et al. 2020, Ward et al. 2023, Gray et al. 2024) and has become integral to downstream analyses of biodiversity patterns, such as (geometric) morphometrics (Gray et al. 2019, Weisbecker et al. 2021, Evers et al. 2022, Navalón et al. 2022) and finite element analysis (Oldfield et al. 2012, Cox et al. 2015, Mitchell et al. 2025).

Despite the promise of digital 3D biodiversity collections, there continue to be hurdles to the rollout of open-access 3D imagery. For example, external, primarily university-based researchers are major generators of 3D imagery of biodiversity specimens in Australia (Weisbecker et al. 2024). However, perceived disincentives for data sharing continue to persist, because of the reliance on competitive research grants to fund the often labor-intensive process of digitization and fear of being scooped (Hipsley and Sherratt 2019). Such monopolization of data access, in Australia and elsewhere, creates inequities in the research community that disproportionately affect early career and unaffiliated researchers and scientists from low- and middle-income countries (Boyer et al. 2016, Davies et al. 2017, Drew et al. 2017, Hipsley and Sherratt 2019).

Another obstacle in the distribution 3D biodiversity images is the limited capacity of most Australian collections to curate these data, mostly because of resourcing constraints (Weisbecker et al. 2024). As a result, researchers lack the guidance and infrastructure to publish their data and are left to make curation decisions guided by their own resourcing and expertise. This, furthermore, leads to a near total lack of institutional oversight even when researchers are sharing their data, so that collections effectively lose control of these 3D images (Weisbecker et al. 2024).

Fortunately, scientific consensus on data sharing is shifting, and there is an increasing expectation from funding bodies and journals that scientific data—including 3D imagery—be made open access and compliant with the FAIR principles (Wilkinson et al. 2016) of being findable, accessible, interoperable, and reusable (OECD 2007, National Health and Medical Research Council 2019, Australian Research Council 2022, Foundation 2023, European Research Council 2024, Nature Portfolio Journals 2024, The Royal Society 2024, Science Journals 2024). With robust policy frameworks, sufficient infrastructure, and user-friendly implementation, researchers will likely adopt FAIR 3D data sharing in a similar way as previously implemented with genetic data through initiatives such as GenBank (Benson et al. 2015) or the International Nucleotide Sequence Database Collaboration (http://insdc.org/documents).

Although FAIR research is a key goal in an equitable research culture, project-based 3D digitization by individuals for the purpose of specific scientific studies cannot progress biodiversity digitization in a way that maximizes the scientific and public benefit of collections. Digitization initiatives are needed that prioritize the collection of 3D imagery to service the needs of diverse stakeholders; these will be termed *digital service collections* in the present article. Digital service collections are already well advanced in some parts of the world (Thiers et al. 2016, Le Bras et al. 2017, Harvard University 2018, Scott et al. 2019, Berlin 2024, Smithsonian Institution 2024); one of the largest and most successful single 3D imaging initiatives is the National Science Foundation (NSF)–funded oVert project, which provides global access to over 29,000 high-fidelity 3D models representing more than 13,000 specimens from 50 institutions (Blackburn et al. 2024). In Australia, however, there is no local precedent for creating, storing, or curating such a multi-institutional service collection of 3D imagery. One of the reasons for this shortfall is the lack of consensus and frameworks for addressing a variety of concerns, ranging from practical considerations such as metadata curation to intellectual property and copyright arrangements that minimize legal risk and balance the needs of institutions and collections users (Davies et al. 2017, Hipsley and Sherratt 2019, Matsui and Kimura 2022, Weisbecker et al. 2024). Furthermore, obtaining funding to support initiatives such as digital service collections is challenging in the current Australian research funding system, leading to a lack of precedents on which frameworks could be developed and refined.

Addressing these policy and infrastructure challenges is an urgent issue because Australian biodiversity is of substantial interest internationally. Australian flora and fauna are highly endemic and an important part of the global story of evolution and diversity of life (Dickman 2018). Australia also leads the world in vertebrate extinctions (Woinarski et al. 2019), and therefore, Australian collections play an especially important role as repositories of historical biodiversity data. FAIR 3D imagery would provide more equitable and diverse community access (Drew et al. 2017, Johnson et al. 2023, Salomon et al. 2023) to these Australian collections while preserving the research mandates of curating institutions. It is therefore critical that planning and policies for data acquisition, storage, and integration with the digital extended specimen data in Australia keep pace with global developments in 3D imaging.

In the present article, we introduce Ozboneviz, an open-access collection of high-fidelity 3D imagery of large Australasian vertebrates hosted on the MorphoSource.org (Boyer et al. 2016) repository. Funded by the Australian Research Council Centre of Excellence for Australian Biodiversity and Heritage, the aims of our project were twofold: to create a digital 3D service collection that elevates research capacity into Australasian land vertebrate diversity (i.e., the clade of tetrapods excluding fishes), with particular focus on larger-bodied mammals of zooarchaeological relevance; and to implement a multi-institutional Australian precedent addressing the challenges of providing open and FAIR 3D biodiversity imagery. In the present article, we describe the project design, status, and community use of the collection. We discuss the implementation of FAIR principles to these data and the usefulness of digital service collections, such as ours, to the international research community, as well as benefits to diverse stakeholders and curating institutions.

We explicitly focus in the present article on FAIR principles as they relate to biodiversity collections, as opposed to the digitization of cultural heritage objects, which have historically been treated separately. However, specimens in Australian biodiversity collections have been widely removed from Country without the knowledge of or consultation with Aboriginal and Torres Strait Islander custodians, even though objects frequently housed in biodiversity collections can hold cultural significance just as much those designated as heritage objects (e.g., Andrews 2017, Ward et al. 2025). It will therefore be desirable for digital biodiversity collections to also be subject to (and, if required, restricted by) future implementations of CARE (collective benefit, authority to control, responsibility, ethics) principles for Indigenous data sovereignty (Carroll et al. 2020).

## Collection contents

**Figure 1.**
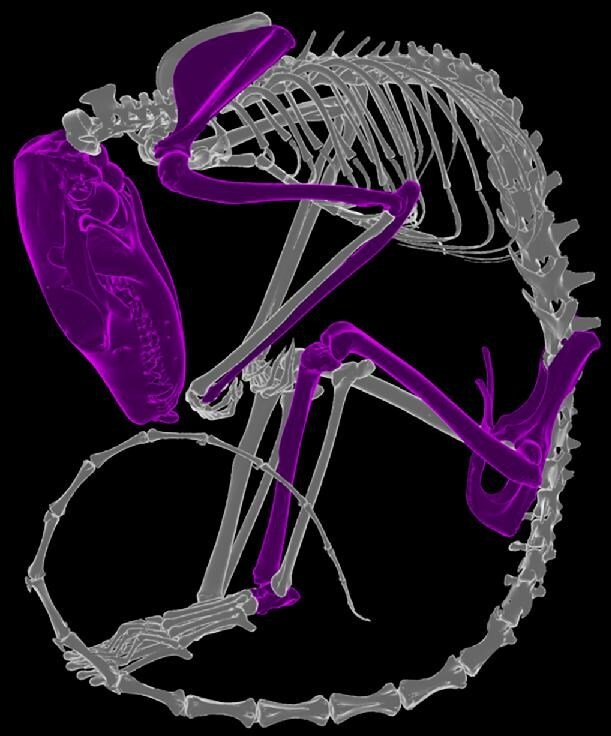
Skeletal elements from the mammal and reptile skeleton digitized by the Ozboneviz project highlighted on a brush-tailed phascogale (*Phascogale tapoatafa*) specimen (SAMA-M3824).

At the time of writing, the Ozboneviz collection (www.morphosource.org/projects/000394988) contains 1591 3D meshes of individual skeletal elements and 46 microcomputed tomography (µCT) images, focusing on the skull and eight major elements from the appendicular skeleton for each species (figure 1; see the supplemental material for our methods). The focus was particularly on mammals of zooarchaeological relevance, which are particularly relevant for interpreting Australia's archaeological record (Mein and Manne 2021). However, species of particular biodiversity value (e.g., the unique marsupial mole) and a representative selection of large reptiles, frogs, and birds were also sampled. Our choice of skeletal elements was determined through balancing our time and budget with stakeholder input as to which elements are most taxonomically informative (e.g., skulls), well preserved in archaeological deposits (e.g., ankle bones), or informative about a species’ skeletal adaptations (e.g., limb long bones and girdles). Ozboneviz excludes fishes, which are covered in the Fishboneviz database (Lambrides et al. 2024).

**Figure 2.**
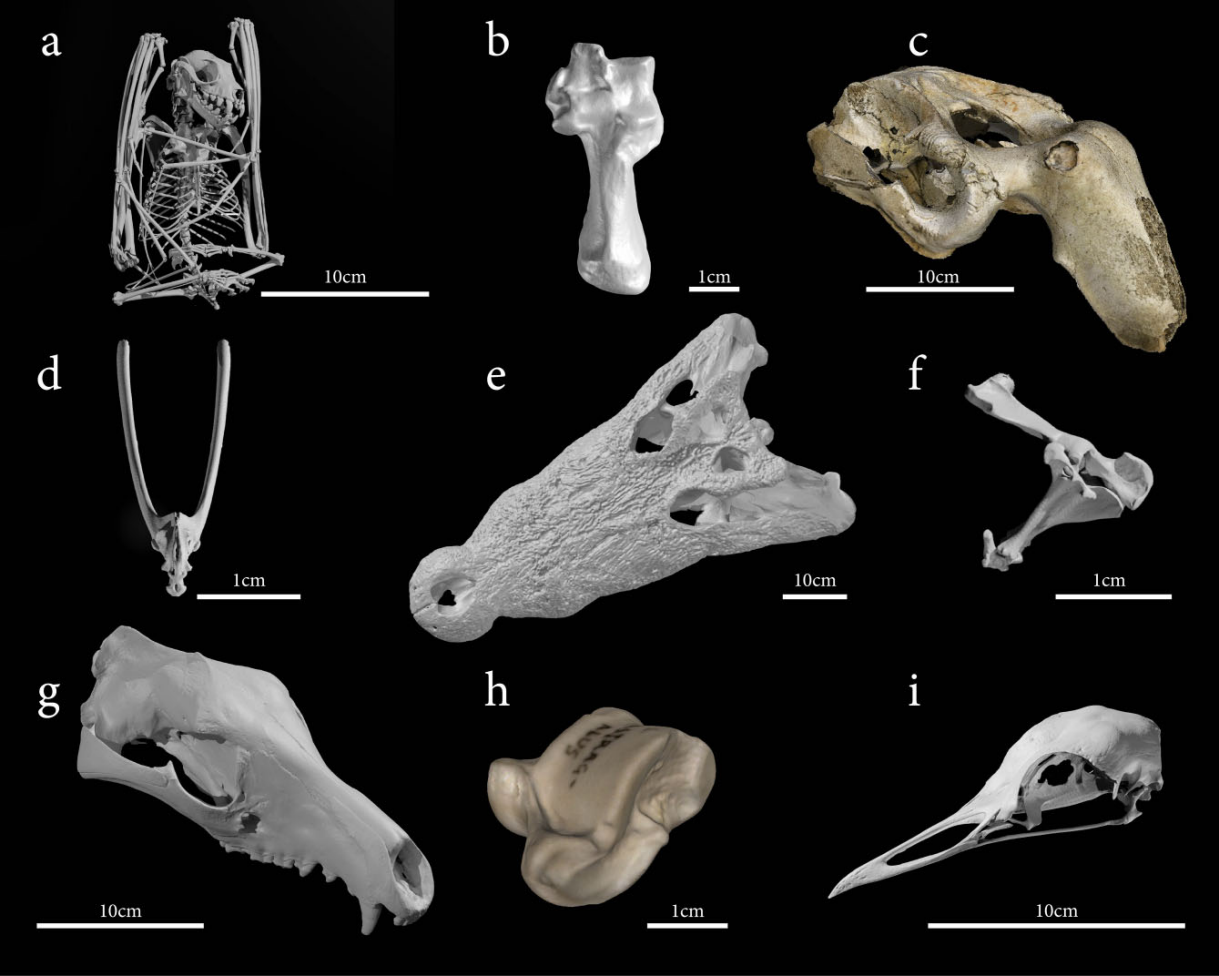
Selection of skeletal elements from the Ozboneviz collection. (a) New Guinea naked-backed fruit bat (*Dobsonia magna*) skeleton (μCT), SAMA-M10475; (b) red-necked wallaby (*Notamacropus rufogriseus*) calcaneus (structured light surface scan), SAMA-M16370; (c) dugong (*Dugong dugon*) cranium (photogrammetry), UQ-257; (d) Australian green tree frog (*Litoria cearula*) pelvis (μCT sourced from MorphoSource), UF-43434; (e) saltwater crocodile (*Crocodylus porosus*) cranium (structured light surface scan), MAGNT-R38573; (f) kakarratul (*Notoryctes caurinus*) hindlimb, (μCT) SAMA-M3139; (g) thylacine (*Thylacinus cynocephalus*) cranium (structured light surface scan), SAMA-M95; (h) goat (*Capra hircus*) astragalus (structured light surface scan), UQ-183; (i) Australian bustard (*Ardeotis australis*) cranium (structured light surface scan), ANWC-B19571.

**Figure 3.**
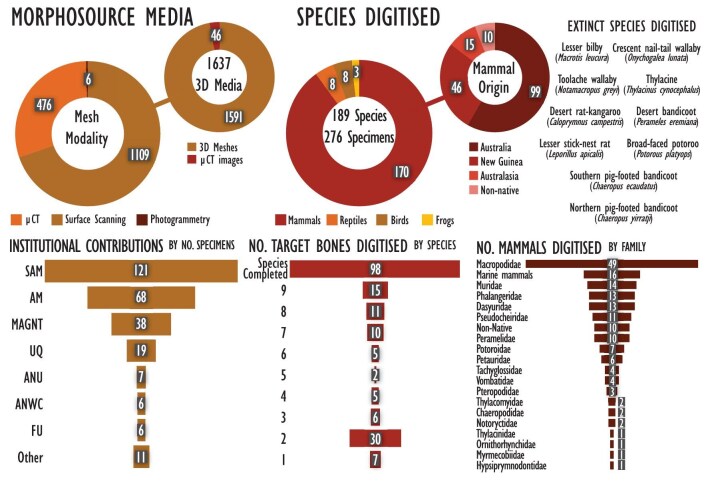
Summary of the Ozboneviz collection 3D imagery acquisition as of 31 March 2025.

We used three 3D imaging modalities (see also Hassett 2018): structured light surface scanning (*n* = 1109), µCT of dry and wet specimens (*n* = 476 individual elements) and photogrammetry (*n* = 6). For a small sample of digitizations using different modalities, see figure 2. A total of 276 individual specimens were imaged, representing 189 tetrapod (land vertebrate) species. Because our budget for µCT scanning and subsequent mesh segmentation was limited, we prioritized the less expensive modality of surface scanning of dry, disarticulated skeletal specimens. We digitized a total of 170 mammal species from Australia and New Guinea, including five monotremes, 122 marsupials, and 43 placental mammals (including non-native and marine mammals; figure 3). We also digitized eight birds, eight reptiles and three amphibians. We acquired 3D imagery of 10 extinct species (figure 3), including digitized elements from a paratype of the extinct northern pig-footed bandicoot (*Chaeropus yirratji* SAMA-M3971), as well as the holotype of golden-mantled tree-kangaroo (*Dendrolagus goodfellowi pulcherrimus* AM-M.21717). In five cases, we digitized captive specimens that we deemed important additions to the collection, because wild representatives of the species were either not available or had insufficient metadata. Organizations that contributed over 10 specimens to the project included the South Australian Museum (*n* = 121), the Australian Museum (*n* = 68), the Museum and Art Gallery of the Northern Territory (*n* = 38), and the University of Queensland (*n* = 19). For more acquisition details and decision-making, see the supplemental material and for a list of specimens, scan modality, and wet versus dry condition (see supplemental table S1).

### Open access, FAIR 3D imagery

We chose the NSF-funded MorphoSource platform to deposit our collection, because this was most aligned with our aim to make Ozboneviz data as FAIR (see above; Wilkinson et al. 2016) as possible. The architecture of MorphoSource allows for connections between 3D data and specimen metadata to be maintained, as well as supporting metadata describing imagery acquisition, processing, intellectual property, and copyright. It also allows data managers to authorize user access to 3D data and track data downloads, intended usage, and user demographics. The platform is also becoming the preferred solution for 3D data storage by US institutions (Blackburn et al. 2024) and provides the facility for collections managers to control of the long-term management of 3D specimen imagery (Boyer et al. 2016), making it internationally compatible. The institutional uptake and facilitation by MorphoSource also ensure these data are likely to remain managed and accurate into the future.

FAIR data are defined as findable by human and machine, have a unique and persistent identifier, are richly described by metadata, and are openly accessible with appropriate authentication protocols where necessary (Wilkinson et al. 2016, Carroll et al. 2020, Hardisty et al. 2022, Sterner and Elliott^ 2024^). FAIR data (and metadata) should also be reusable and should have clearly defined parameters for reuse. Following these requirements, researchers external to our project group have minted 92 DOIs for our 3D imagery on MorphoSource. In addition, 211 specimens are linked to an occurrence record on iDigBio (Integrated Digitized Biocollections, www.idigbio.org), which allows the specimen metadata on MorphoSource to remain synchronized with the institutional metadata over time.

All the Ozboneviz collection is freely available to be downloaded and reused by registering a user account with MorphoSource. Conditions for reuse of these 3D data are clearly stated in the CC-BY-NC copyright policy and standard MorphoSource agreement. Users must agree to these conditions and receive a copy of the license and usage agreement when downloading each media. Note also that copyright conditions can be changed to suit the managing collections.

## Community usage

**Figure 4.**
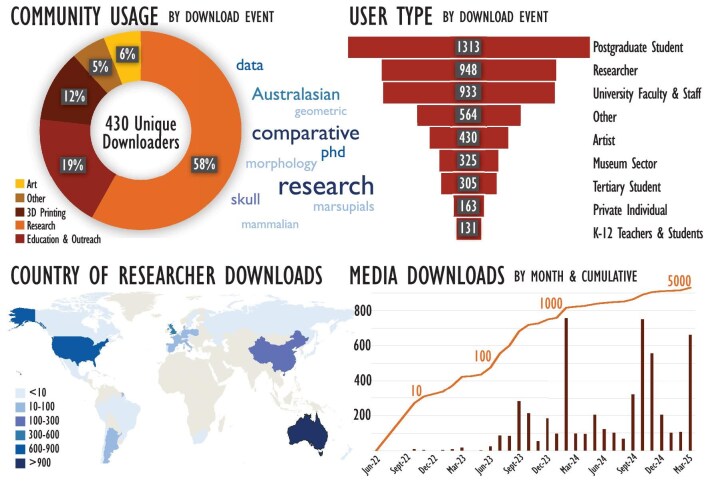
Summary of community usage of the Ozboneviz collection as of 31 March 2025.

MorphoSource captures information on the proposed use and demographics of users as a condition of downloading data. These data are self-reported by users who can select multiple options to describe themselves and their proposed data use. As of March 2025, 1269 individual models have been downloaded a total of 5237 times. The most common reported usage of these data is for research (58%), followed by education and outreach (19%) and 3D printing (12%; figure 4). Users who identified as postgraduate students are the largest downloaders of our data. Educators, students, and university-based researchers from institutions in North America, South America, Europe, Asia, Africa, and Australasia have downloaded our 3D imagery a total of 3033 times (figure 4). A small but unexpected use of these 3D imagery has been by artists either as reference models for traditional analogue art practices (drawing, modelling, etc.) or for use in digital art practices. To date, our four most downloaded 3D media are a platypus cranium (*Ornithorhynchus anatinus*), saltwater crocodile cranium (*Crocodylus porosus*), and thylacine (*Thylacinus cynocephalus*) cranium and mandible. Intriguingly, the platypus and a crocodilian (the gharial) were also reported as the popular downloads of the oVert projects (Blackburn et al. 2024). In our case, downloads were to equal measure for research and outreach, which might reflect the fact that these two species are among Australia's most distinctive and evolutionarily unique endemics.

## Technical challenges

We encountered several challenges in assembling the Ozboneviz collection related to specimen suitability for imaging and specimen metadata. Our project goal was to digitize 8–10 bones per species (depending on taxonomic class), and our core acquisition modality was surface scanning of dry, skeletonized specimens. However, digitizing all our target bones for each species was more challenging than anticipated, even where we were able to digitize bones from multiple individuals of the same species. Postcranial skeletal elements are less frequently preserved in Australian biodiversity collections than skulls or were often unsuitable for surface scanning, because of adhering soft tissue, translucency caused by bone grease, or articulation with neighboring bones. We successfully digitized all target bones for 104 species, although 50 species have only five or fewer bones digitized (figure 3). We also found that the completeness of specimen metadata varied widely because of historical realities of collections management. For example, many specimens were provided as roadkill were too decayed to provide a sex, which can be important information for downstream analyses. In these cases, we prioritized specimen intactness and our ability to produce high-quality 3D imagery over metadata completeness.

The extensive use of µCT for full skeleton acquisitions was not within the scope of our funding, but we were able to choose a selection of particularly rare or important species for full-body µCT acquisitions (see the supplemental material for additional challenges specific to this modality). Non-native and domestic animals were also an important component of our collection but are often not curated in Australian museum biodiversity collections. We primarily relied on university-based collections for placental mammals such as foxes, cats, dogs, horses, and domestic artiodactyls. However, we found that specimen metadata in university collections was generally poorer than museum-based collections. Unlike the United States, where many universities maintain museums with internationally recognized collections, most Australian university collections have a focus on teaching and informal referencing. For example, even though most specimens have accession numbers and clear governance frameworks, Australia's major zoological collections aggregator, the Online Zoological Collections of Australian Museums (OZCAM), explicitly excludes university collections. Similarly, the only Australian university collections represented on the Global Biodiversity Information Facility (GBIF, gbif.org) are contributors to the Australasian Virtual Herbarium initiative and the University of Adelaide's Waite Insect and Nematode collection.

### Permission and legal framework challenges

We are not aware of a clear interpretation of Australian copyright and intellectual property laws on the legal status of 3D biodiversity specimen imagery and IP ownership of scans (Weisbecker et al. 2024)—for example, as provided for the United States by D’Andrea and colleagues (2022). The sector is therefore missing clear guidance on key issues; for example, on whether 3D images of objects produced by nature have any default copyright protection, which is not the case in the United States (D'Andrea et al. 2022). Because of this legal vacuum and the lack of an Australian precedent, it was particularly challenging to identify appropriate terms under which specimen imagery could be acquired and published to the satisfaction of participating institutions. The core team engaged in extensive consultation with collection representatives to discuss diverse institutional perspectives on issues such as scan ownership, intellectual property, and copyright. For this purpose, our team developed an FAQ document with support from the MorphoSource Team. Although all individuals we approached were supportive of the concept of Ozboneviz, the relative novelty of the digital service collection model made it difficult to assess the copyright implications and financial risk, and it was not always possible to find clear permission pathways. Therefore, some collections felt unable to issue a permission for data acquisition. The South Australian Museum issued a formal permission text, which became a useful basis for permissions from other collections. However, the lack of easily accessible interpretation of copyright law on the matter of 3D biodiversity images will likely remain a substantial hindrance for the sector.

## Ozboneviz as a digital service collection for Australian vertebrate skeletal 3D imagery

Ozboneviz has succeeded in providing a comprehensive 3D skeletal database of Australian land vertebrates. We see it as a timely contribution in the continuously expanding landscape of 3D biodiversity data, on the basis of the enthusiastic adoption by the scientific and wider community.

Our usage data show that the initial scope of Ozboneviz as a resource for zooarchaeological data was quickly expanded into other areas of science (such as paleontology and evolutionary biology), public engagement, and the arts. This is an excellent example of how the availability of open-access data can have unforeseen benefits well beyond the original scope of the data collection if it is made accessible in appropriate ways (Suarez and Tsutsui 2004, Davies et al. 2017, Lendemer et al. 2020, Blackburn et al. 2024). In addition, despite its novelty, our download statistics reveal that several international research efforts are using Ozboneviz data, demonstrating the almost immediate positive impact of the collection in representing Australian vertebrate diversity to the international research community.

As was outlined in the introduction, one of the greatest potential benefits of open-access 3D data is the improvement in equitability of access to researchers and other stakeholders (Cook et al. 2014, Drew et al. 2017, Hipsley and Sherratt 2019). The urgency of this is particularly stark in our user statistics, with by far the greatest download activity coming from the most junior researcher cohort—postgraduate students. The appetite for these data is clearly substantial among these early-career researchers, who are often the most disadvantaged in terms of the funding, time, and reputation required to individually access these specimens. In addition, nearly 20% of our downloads are reported for education or outreach, and a sizeable proportion of the users are tertiary or school students and teachers. This demonstrates the excellent use of Ozboneviz to provide a novel means for the general public to access and interact with Australia's museum collections.

The volume of downloads also emphasizes the usefulness of 3D digital service collections to the curating institutions that publish these data. Once digitized and published as an open download, there is no further need for additional staffing or specimen handling, whereas the download volume far outstrips the capacity of collection managers to facilitate an equivalent rate of access to physical specimens. Because specimen downloads are monitored and usage is aggregated by MorphoSource, this expanded impact can be measured easily and is valuable for demonstrating the collection impact.

Although the majority of the Ozboneviz collection came from our own digitizations, we incorporated data from other open-access MorphoSource collections—mostly from the oVert project collections network—to improve our coverage of species. This highlights the benefit of using the MorphoSource platform, which allows projects such as ours to derive new 3D imagery from published media in other collections and therefore enhance the impact of an individual vouchered specimen (Blackburn et al. 2024). This can fill gaps in collections, as was the case for Ozboneviz, but it is also possible to create entirely new collections just from derivatives of other collection data. An example of this is the Fishboneviz collection (Lambrides et al. 2024), a sister project to Ozboneviz that relied purely on segmentation of already published CT scans to generate an 3D reference collection for Australian and Pacific Ocean fishes of zooarchaeological importance.

## Ozboneviz as a precedent for Australian open-access 3D biodiversity data

An important objective for Ozboneviz was the generation of a test case that would demonstrate the feasibility and clarify the challenges of open-access 3D biodiversity data in Australia. Our hope is that the success of this collection will inform a broader conversation about the value and management of open-access FAIR 3D biodiversity imagery in the future. Many of the challenges we encountered reflect the issues raised in a submission by the National Imaging Facility Museums and Collections Special Interest Group to the Accessing Australia's Research Collections stakeholder consultation by the Australian Academy of Science (Weisbecker et al. 2024).

Appropriate archival storage for 3D imagery that also facilitates data discoverability and retrieval is currently an urgent challenge faced by curating institutions (Atlas of Living Australia et al. 2023). Large file sizes and secure archival storage have long been a problem for 3D imagery producers and users (Rowe and Frank 2011, Boyer et al. 2016). Collections managers are well versed in 3D imaging and recognize its value in collections preservation, data dissemination, research efficiency, and public outreach (Schindel and Cook 2018, Hilton et al. 2021). However, these frontline managers cannot curate the large volumes of 3D imagery already being generated from their collections without institutionally supported storage and data management solutions (Weisbecker et al. 2024). This means these data, often acquired at high cost using competitive taxpayer-funded grants, are effectively lost to the institution and wider research collection unless deposited on external data repositories (Davies et al. 2017, Hipsley and Sherratt 2019, Lewis 2019). The Ozboneviz initiative addressed this issue by identifying a digitization strategy for a service collection that addressed a particular priority (in this case, zooarchaeology and land vertebrate diversity). This made it a sufficiently discrete work package to attract funding and addressed both the issue of storage and of high-quality presentation, with comparatively extensive longevity (the funding covers 14 years of MorphoSource storage and presentation). It is therefore a useful blueprint for further digitization in Australia's fragmented funding landscape.

It is important to note that the use of MorphoSource by Ozboneviz formalizes an existing informal Australian trend of extensive deposition of 3D data. As of March 2025, excluding the Ozboneviz collection, 2642 3D media representing 2766 specimens held by Australian-based museums are currently stored on MorphoSource. Of these, 73% are published as open access, but in most cases, the managing organization is not the collection from which the specimens were digitized. In establishing the Ozboneviz collection, we recognized that Australian collections managers are not yet resourced to manage these data but hope that it will demonstrate the feasibility of a 3D digital collections approach and therefore encourage investment into the necessary infrastructure. Our goal is to transfer management of the Ozboneviz 3D imagery to the contributing institutions as soon as practicable.

## Opportunities, risks, and limitations of implementing collections such as Ozboneviz

Online repositories provide a technological solution to 3D data storage, but a range of concerns were frequently raised throughout our project. A major issue is the fact that both the storage and the presentation platform for Ozboneviz are overseas, in the United States. There are valid concerns about the risks associated with the storage of digital specimen data on servers outside of Australia and external to the governance structures of the curating institutions. For example, many curating institutions that contributed specimens to Ozboneviz are state government agencies within Australia, but data uploaded to MorphoSource are subject to US laws (MorphoSource 2024). This could be overcome by connecting Australian server space to the MorphoSource platform or even by hosting an Australian-governed MorphoSource instance that integrates with the global MorphoSource interface.

Another issue remains the assessment of risk in the provision of open-access 3D data—for example, that 3D images might be monetized by private individuals (Watanabe 2018, Matsui and Kimura 2022). In the context of the large volume of 3D data generated by open-access initiatives such as oVert, this risk remains undemonstrated and may be negligible. However, as a means of managing such risks, MorphoSource offers a range of options for data licensing, intellectual property assignment, and data management control.

Although these concerns are valid, it must be acknowledged that the process of making data more open and more FAIR will also likely coincide with some increased risk. We also highlight that 3D imagery are just one form of collections-derived data. Within the context of Australian biodiversity collections, there is ample precedent for accepting and managing such risks in the routine publication of other data modalities by collections such as occurrence data (Commonwealth Scientific and Industrial Research Organisation 2024) and two-dimensional photographic imagery (Australian National Botanic Gardens and Australian National Herbarium 2024) or genetic sequence data (Benson et al. 2015) by researchers. Ultimately, given the value of biodiversity collections in combating urgent global scale threats such as biodiversity loss, we argue that the risks are outweighed by the benefits of scientific capacity building as our results already demonstrate.

Broadly, the linkability and discoverability of collections-derived imagery are another challenge in creating Australian 3D biodiversity service collections. For example, Ozboneviz imagery published to MorphoSource does not directly link to any platforms managed by the curating institutions or vice versa. Currently, MorphoSource can link digitized specimens to the United States–based iDigBio aggregator, and 3D specimen imagery is discoverable via the GBIF biodiversity aggregator if museums include links to the MorphoSource entries in their databases. However, links are missing to key biodiversity data aggregators such as OZCAM (Wallis 2006) or the Atlas of Living Australia (Australian National Botanic Gardens and Australian National Herbarium 2024, Council of Heads of Australian Faunal Collections 2024). As with the provision of server space, this is an infrastructure issue that could be addressed by a separate, targeted initiative in the future to leverage the impact of 3D service and research collections. It is also worth highlighting that improving linkage and interoperability are core to initiatives such as the digital extended specimen network (Hardisty et al. 2022), but the institutions that Ozboneviz collaborated with are not yet a part of such initiatives. However, MorphoSource is positioned well to grow these capabilities in the future through features such as the recently published MorphoSource Terms Vocabulary (MorphoSource 2024), which uses Darwin Core (Wieczorek et al. 2012) to reference specimens in collections in line with other repositories. As custodians of the source material for 3D biodiversity imagery, clear leadership, policies, and guidelines from decision-makers within curating institutions regarding imagery acquisition and data sharing will be crucial to establish an optimized and sustainable, open-access 3D data sharing culture in Australia.

As was noted in the introduction, it is also important to recognize that, despite not housing cultural heritage objects, biodiversity collections represent the removal of plants and animals from Country and the custodianship of Indigenous peoples and is intimately tied to the extractive economics of colonization and privileging of Western epistemology and values (De Vos 2007, Mackenzie 2017, Weber 2021, Ward et al. 2025). The Ozboneviz collection does not and could not represent an attempt to rectify this situation. Nor do we suggest that access to 3D imagery should stand in lieu of repatriation of culturally significant objects to Indigenous communities affected by colonial collecting practices. Further investment and consultation are needed to ensure that FAIR 3D imagery initiatives also meet the CARE principles for Indigenous data sovereignty (Carroll et al. 2020) to address the concerns and needs of Indigenous communities.

Our implementation of Ozboneviz has shown that digital service collections of 3D imagery are feasible and come with substantial benefits to the general and scientific public, giving them an important place in the future of Australia's biodiversity collection sector. Our initiative has also identified clear limitations such as storage, presentation, and data security concerns, which all require separate—likely long-term—efforts to address. However, the successful rollout of similar—and larger—initiatives in the United States shows that these issues are surmountable. The use of MorphoSource storage and discrete digital service collections such as Ozboneviz are then a useful and safe avenue for managing the increasing data volumes following best practices while suitable frameworks are being developed.

## Supplementary Material

biaf064_Supplemental_Files

## Author Biography

Vera Weisbecker (vera.weisbecker@flinders.edu.au) is an evolutionary biologist and Erin Mein (erin.mein@flinders.edu.au) is an archaeologist postdoctoral researcher at the College of Science and Engineering, Flinders University and the Australian Research Council Centre of Excellence for Australian Biodiversity and Heritage (CABAH), in Adelaide, South Australia, Australia. Diana Fusco is a palaeontologist and research assistant and Jacob van Zoelen is a PhD student and research associate at the College of Science and Engineering, Flinders University and CABAH, in Adelaide, South Australia, Australia. Sandy Ingleby is a zoologist and Collection Manager of the Mammal collection at the Australian Museum, in Sydney, New South Wales, Australia. Ariana Lambrides is an archaeologist at the College of Arts, Society and Education, James Cook University, in Cairns, Queensland, Australia. Tiina Manne is an archaeologist at the School of Social Science, the University of Queensland in Brisbane, Queensland, Australia. Keith Maguire is Head of the Natural Science Collections at the South Australian Museum, in Adelaide, South Australia, Australia. Sue O'Connor is an archaeologist at the College of Asia and the Pacific, Australian National University and CABAH, in Canberra, Australian Capital Territory, Australia. Thomas J. Peachey is Technical Officer of 3D Digitisation at the Australian Museum, in Sydney, New South Wales, Australia. Sofia C. Samper Carro is an archaeologist at the College of Asia and the Pacific, Australian National University and CABAH, in Canberra, Australian Capital Territory, Australia. David Stemmer is a zoologist and Collection Manager at the South Australian Museum, in Adelaide, South Australia, Australia. Jorgo Ristevski is a palaeontologist and postdoctoral researcher at the Max Planck Institute of Geoanthropology in Jena, Germany and Griffith University in Brisbane, Queensland, Australia. Pietro Viacava is the Digital Research Curator of the Australian National Wildlife Collection, Commonwealth Scientific and Industrial Research Organisation, in Canberra, Australian Capital Territory, Australia. Adam M. Yates is Senior Curator of Earth Sciences at the Museum and Art Gallery of the Northern Territory, in Alice Springs, Northern Territory, Australia.
