# Supplementary material for: Ozboneviz: an Australian precedent in FAIR 3D imagery and extended biodiversity collections
Source: Bioscience. 2025 Jun 10;75(9):747–56. doi: 10.1093/biosci/biaf064 (PMC12412300; doi:10.1093/biosci/biaf064)
Supplement: biaf064_Supplemental_Files [file biaf064_supplemental_files.zip › Ozboneviz_supplementary_methods_R1.docx]

## **Supplementary Methods**

The Ozboneviz digitisation initiative was a collaboration of a network of stakeholders. It consists of a core team of project managers and digitisation officers, as well as contributing staff within collections who facilitated access, co-acquired data, and supported the process of acquiring permissions. The core team included the project lead (Vera Weisbecker), several academic stakeholder-advisors, the equivalent of one year’s full-time position of a project manager and chief acquisition officer (Pietro Viacava followed by Jacob van Zoelen), supported by several casual and short-term project officers tasked with database management, 3D image acquisition, segmentation and data upload (Erin Mein, Diana Fusco and Jorgo Ristevski). Funding for these positions was provided by the Australian Research Council Centre of Excellence for Australian Biodiversity and Heritage (CABAH) through a Legacy Grant Scheme.

In consultation with stakeholders and project collaborators, we developed a target list of 130 Australian mammals, 28 New Guinean mammals, 11 birds, 36 reptiles and 5 frogs. We prioritised digitising the skull and eight major elements from the appendicular skeleton for each species. Mammalian and reptilian postcranial bones consisted of the scapula, humerus, ulna, pelvis, femur, tibia, astragalus (talus) and calcaneum (calcaneus) (Figure 1 of the manuscript). For birds we digitised eight bones including the coracoid-scapula, carpometacarpus, tibiotarsus and tarsometatarsus. For frogs we aimed to digitise 10 bones including the coracoid and calcaneum. Skeletal elements such as vertebrae, phalanges and ribs which are numerous and therefore, less optimal for estimating minimum numbers of individuals in palaeozoological contexts, were not digitised. In the majority of cases, we chose not to digitise the radius or fibula, although some exceptions included bats (Chiroptera), birds (Aves), frogs (Anura) and artiodactyls (Artiodactyla) where these elements are either taxonomically distinctive or fused to the ulna or tibia. Specimens were digitised from a range of museums (a breakdown is in Table 1).

**Table 1** Number of specimens digitised by curating institution. *CT imaged specimens sourced from oVert collaborating institutions via MorphoSource.

| **Curating Institution** | **No. Specimens** |
| --- | --- |
| South Australian Museum | 121 |
| Australian Museum | 68 |
| Museum and Art Gallery of the Northern Territory | 38 |
| The University of Queensland | 19 |
| The Australian National University | 7 |
| Australian National Wildlife Collection | 6 |
| Flinders University Palaeontology Laboratory | 6 |
| California Academy of Sciences* | 2 |
| Laboratory of Emma Sherratt, University of Adelaide | 2 |
| Michigan State University Museum | 2 |
| University of Florida, Florida Museum of Natural History* | 2 |
| Harvard University Museum of Comparative Zoology* | 1 |
| Museum of Vertebrate Zoology, UC Berkeley* | 1 |
| Museum of Zoology, University of Cambridge | 1 |

##### *Biodiversity collections*

We digitised vouchered specimens from Australian-based biodiversity collections at the South Australian Museum, Museum and Art Gallery of the Northern Territory, the Australian Museum and the Australian National Wildlife Collection. Specimens from these institutions were prioritised as each have clearly defined public access protocols and specimen metadata is rich, formatted to Darwin Core standards and discoverable via biodiversity data aggregators such as OZCAM (Council of Heads of Australian Faunal Collections, 2024), GBIF (Facility 2025) and iDigBio (iDigBio 2023). Secondary sources of specimens were the University of Queensland Archaeology Laboratories, the Australian National University Zooarchaeology Collection and Flinders University Palaeontology Laboratory. Select specimens that were difficult to source were segmented from computed tomography image stacks deposited on MorphoSource.org by international institutions or private collections. We also received donations of 3D imagery of cetaceans from Dr Toshiyuki Kimura of the Gunma Museum of Natural History (Kimura 2023).

##### *Imaging modalities*

We used three 3D imaging modalities, structured light surface scanning, micro-computed tomography (µCT) and photogrammetry. All µCT was undertaken at the Flinders University Medical Device Research Institute using a Nikon XT H 225ST. As our budget for µCT and subsequent mesh segmentation was limited, we prioritised surface scanning dry, disarticulated skeletal specimens. Structured light scanners were brought to each collection to digitise specimens in-house rather than initiate large numbers of specimen loans. We used multiple structured light surface scanning devices consisting of a Polyga Compact S1, a Solutionix C500, a Shining 3D Einscan Pro+ and an Artec Space Spider. As each has optimal operating conditions the choice of device was determined on a balance of specimen size, collection location, light conditions and project stage. For example, we used the Polyga (point distance = 0.085mm) for very small specimens and the Einscan Pro+ (point distance = 0.24mm with turntable and 0.7mm when handheld) for medium to large specimens in low-light conditions. We acquired the Artec Spider (point distance = 0.15mm) part-way through our digitisation program after which it became the preferred device for digitising medium and large specimens. The Solutionix was located at the University of Queensland and used for digitising specimens from the Archaeology Laboratories. Texture (photorealistic colour) capture was not a priority and not available across all devices but, where possible, was captured to increase the utility of the 3D imagery. Photogrammetry using Agisoft software was only used for few large specimens when the available surface scanning devices were unsuitable or unavailable at the time of scanning.

##### *Specimen selection*

Specimens were selected for digitisation based on a balance between metadata completeness, specimen condition and species rarity. Because our goal was to create a service collection for researchers, it was important that end users could account for potential influences of animal age, sexual dimorphism or geography on skeletal morphology. We therefore aimed to digitise adult, wild specimens that were free from pathologies with metadata on sex and collection location. However, we found the completeness of these metadata varied widely owing to historical realities of collections management. When choosing suitable specimens, we avoided clearly juvenile specimens based on molar eruption and fusion of epiphyses. Known captive specimens (e.g. from zoos) were also avoided when possible, owing to their potential for morphological differences with wild specimens (e.g. Crossley and del Mar Miguélez 2001, Hartstone-Rose et al. 2014, Mitchell et al. 2021). We digitised captive animals only as a last resort after we had exhausted other avenues to source wild specimens. Type specimens were not specifically targeted for digitisation as many are held in collections outside of Australia but, were treated as high priority where available.

Computed tomography was used for particularly rare and important species, which also allowed us to image many whole animals as wet specimens (such as the brush-tailed phascogale in main manuscript Fig. 1), rather than disarticulated bones. This presented its own challenges; in some cases, metadata did not record details on specimen sampling and preparation methods that can impact on digital imaging. For example, roadkill specimens can have substantial damage to the skeleton that is unable to be ascertained prior to µCT (Figure 1). Historical specimens of bats and birds were once commonly collected via shooting. In the New Guinea naked-backed fruit bat specimen we found embedded lead shot pellets that produced substantial noise in the computed tomography images and made the segmentation of individual bones difficult. In some cases, identifying where the skull of fluid-preserved specimens had been removed and replaced with foam formers was difficult and resulted in additional imaging costs and time spent by our team and curatorial staff to identify and reimage the head separately (such as the extremely rare marsupial mole *Notoryctes caurinus*) or more appropriate specimens. However, the reuse value of the µCT volume stacks of these ‘headless’ or damaged specimens is high, and we chose to make these data available on MorphoSource as well. These instances highlight the vital importance of metadata discoverability to collections users and the need to resource curatorial staff with the appropriate tools that allow for specimen lifecycle traceability and digitally catalogued links between all data points of the Digital Extended Specimen (Hardisty et al. 2022, Lendemer et al. 2020, Webster 2017).


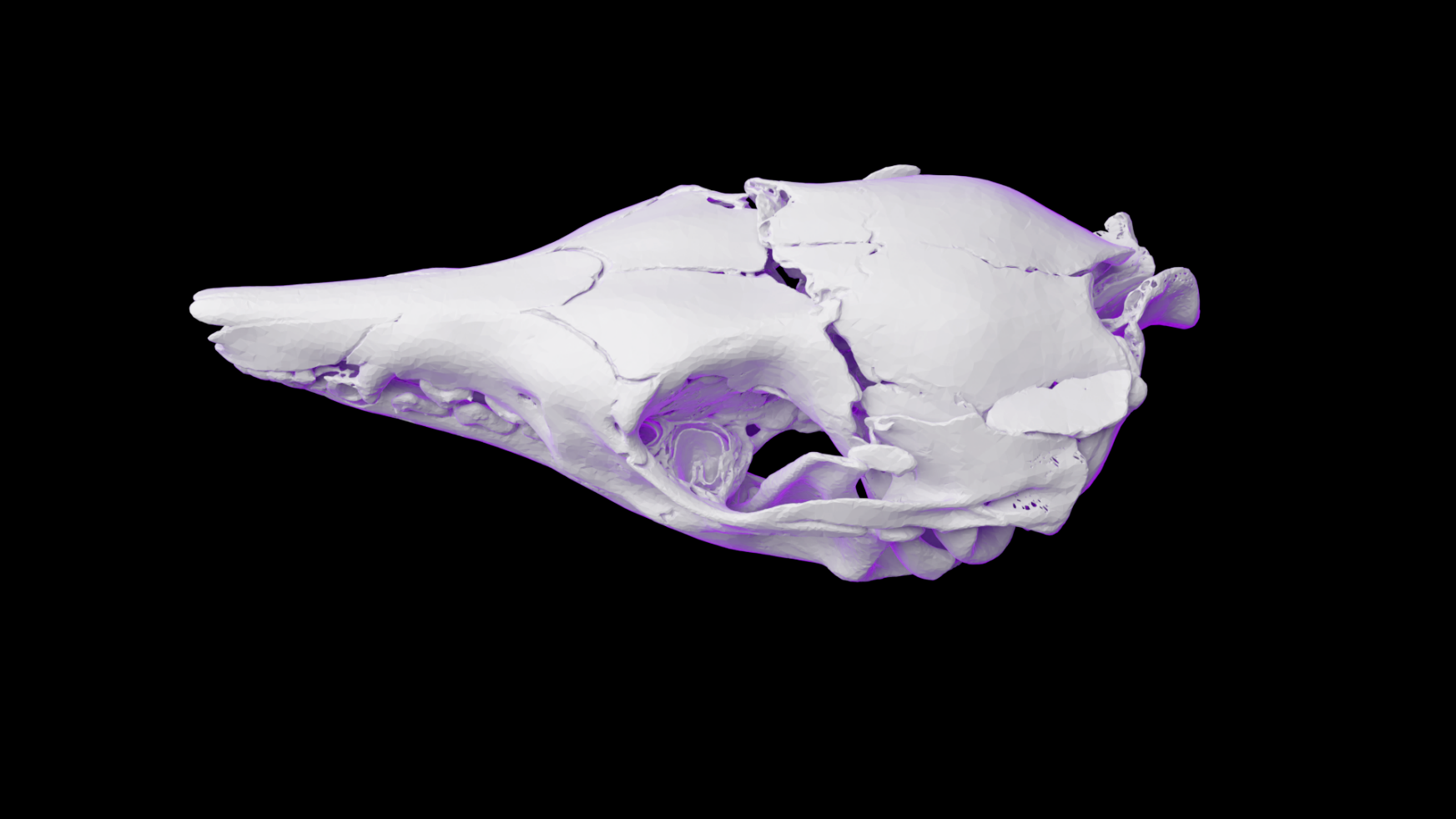


**Figure 1** Damaged skull of a greater bilby (*Macrotis lagotis*) revealed after µCT imaging. This specimen (SAMA-M3600) was unsuitable for creating 3D models for the Ozboneviz collection but the µCT volume stacks are made available on MorphoSource.org.

##### *3D image generation*

3D point clouds acquired via structured light scanners were meshed using the proprietary software associated with each scanning device. This included Flexscan3D (Polyga 2021) ezScan2017 (Solutionix) (Medit 2019), Einscan Pro v2.6.0 (Shining 2022) and Artec Studio 17 Pro (Artec 2022). The 10 target skeletal elements were segmented from µCT volumetric image stacks using Materialise Mimics v. 24 (Materialise 2022) and 3D Slicer (Fedorov et al. 2012). We used Agisoft Metashape v.1.8.2 (Agisoft 2022) to generate 3D meshes from digital photographs. 3D meshes were exported as .PLY format files or Wavefront .OBJ files if texture was captured. We avoided making extensive modifications to the exported meshes but in some cases filled small holes or smoothed meshes in Geomagic Wrap v2021.2.2 (3D Systems 2021) while noting these modifications in the file metadata.

##### *Data sharing*

We purchased storage on the [MorphoSource.org](http://www.MorphoSource.org) platform because it has been designed as a FAIR 3D data archive that balances the needs of data producers, users and curating institutions (Boyer et al. 2016). The architecture of the MorphoSource repository allows for connections between 3D data and specimen metadata to be maintained, as well as supporting metadata describing imagery acquisition, processing, intellectual property and copyright. MorphoSource also has the capacity for data managers and curating institutions to authorise user access to 3D data and track data downloads, intended usage and user demographics. We uploaded 3D meshes derived from our three imaging modalities to MorphoSource alongside digital photographs of the physical specimen and any catalogue tags or accompanying documentation to maximise information on specimen condition and provenance. We also uploaded all volumetric image stacks produced via µCT and linked these as ‘Parent Media’ to the 3D meshes of that specimen derived by segmentation. We chose not to upload raw point cloud data owing to the large file sizes and dependence on proprietary software which makes these poor candidates for data archiving. At a minimum all 3D imagery is provenanced to a physical specimen using Darwin Core (Wieczorek et al. 2012) standard terminology for institution, collection, catalogue number and taxonomy and is further described by skeletal element and body side. Where it exists, each 3D image was also linked to a corresponding occurrence record on the iDigBio (iDigBio 2023) biodiversity data aggregator. Although iDigBio ingests data from the Atlas of Living Australia, the facility to link 3D imagery on MorphoSource directly to this Australian based data aggregator is not yet available.

Where taxonomic names imported from iDigBio clash with more recent specimen identifications or taxonomic revisions we made use of the MorphoSource facility to include both taxonomies. All 3D imagery in the Ozboneviz collection has metadata describing imaging modality, device, operator, post-processing interventions that have altered the mesh surface and descriptions of any conditions that may obscure or alter the natural bone morphology such as adhering soft tissue, damage or scanning artefacts. All 3D imagery deposited on MorphoSource are assigned an Archival Resource Key (ARK) as a persistent identifier. MorphoSource also have the capacity to mint Digital Object Identifiers (DOI) and we strongly encourage researchers to request and cite DOI’s for each 3D media they use.

Intellectual property over the 3D imagery is assigned to the curating institutions (i.e. museum or university). All 3D imagery produced by Ozboneviz is provided to users as ‘Open Download’ under a Creative Commons BY-NC 4.0 copyright licence which permits non-commercial use, sharing and adaptation of these data.

**References**

3D Systems. 2021. Geomagic Wrap v2021.2.2.

Agisoft. 2022. Metashape v. 1.8.2.

Artec. 2022. Artec Studio 17 Pro.

Boyer DM, Gunnell GF, Kaufman S, McGeary TM. 2016. Morphosource: archiving and sharing 3-d digital specimen data. The Paleontological Society Papers 22: 157-181.

Crossley DA, del Mar Miguélez M. 2001. Skull size and cheek-tooth length in wild-caught and captive-bred chinchillas. Archives of Oral Biology 46: 919-928.

Facility GBI. 2025. Global Biodiversity Information Facility. (27/03 2025; <www.gbif.org>)

Fedorov A, et al. 2012. 3D Slicer as an Image Computing Platform for the Quantitative Imaging Network. Magnetic Resonance Imaging 30: 1323-1341.

Hardisty AR, et al. 2022. Digital extended specimens: enabling an extensible network of biodiversity data records as integrated digital objects on the internet. BioScience 72: 978-987.

Hartstone-Rose A, Selvey H, Villari JR, Atwell M, Schmidt T. 2014. The three-dimensional morphological effects of captivity. PLOS ONE 9: e113437.

iDigBio. 2023. Integrated Digitized Biocollections. (25/11 2024; <www.idigbio.org>)

Kimura T. 2023. 3D digitization of the natural history museum specimens: A case study of building the 3D digital database of the periotic and tympanic bulla of modern cetaceans (in Japanese). Bulletin of Gunma Museum of Natural History 27: 211-216.

Lendemer J, et al. 2020. The extended specimen network: a strategy to enhance us biodiversity collections, promote research and education. BioScience 70: 23-30.

Materialise. 2022. Mimics v. 24.

Medit. 2019. ezScan 2017: Medit Metrology Group.

Mitchell DR, Wroe S, Ravosa MJ, Menegaz RA. 2021. More challenging diets sustain feeding performance: Applications toward the captive rearing of wildlife. Integrative Organismal Biology 3: obab030.

Polyga. 2021. Flexscan3D.

Shining D. 2022. EinscanPro v2.6.0.9: Shining 3D.

Webster MS. 2017. The extended specimen: emerging frontiers in collections-based ornithological research. CRC Press.

Wieczorek J, Bloom D, Guralnick R, Blum S, Döring M, Giovanni R, Robertson T, Vieglais D. 2012. Darwin Core: An Evolving Community-Developed Biodiversity Data Standard. PLOS ONE 7: 1-8.
