# Supplementary material for: Ozboneviz: an Australian precedent in FAIR 3D imagery and extended biodiversity collections
Source: Bioscience. 2025 Jun 10;75(9):747–56. doi: 10.1093/biosci/biaf064 (PMC12412300; doi:10.1093/biosci/biaf064)
Supplement: biaf064_Supplemental_Files [file biaf064_supplemental_files.zip › Weisbecker_etal_R1_Supp_Table.docx]

| **Taxonomic name** | **Collection Catalogue No.** | **Specimen Preservation** | **Imaging Modality** |
| --- | --- | --- | --- |
| *Aepyprymnus rufescens* | MSU:MR:MR.4680 | Wet | μCT |
| *Aepyprymnus rufescens* | SAMA:Mammalogy:M2939 | Wet | μCT |
| *Aepyprymnus rufescens* | SAMA:Mammalogy:M8051 | Wet | μCT |
| *Antechinus flavipes* | SAMA:mammalogy:M27013 | Wet | μCT |
| *Aquila audax* | UMZC:Vertebrates:508.F | Wet | μCT |
| *Arctocephalus pusillus doriferus* | SAMA:Mammalogy:M15297 | Dry skeletonised | Structured light |
| *Ardeotis australis* | ANWC:Birds:B19571 | Dry skeletonised | Structured light |
| *Ardeotis australis* | MAGNT:Bird:T1084 | Dry skeletonised | Structured light |
| *Berardius arnuxii* | SAMA:Mammalogy:M5012 | Dry skeletonised | Structured light |
| *Bettongia gaimardi* | MAGNT:Mammal:U8056 | Dry skeletonised | Structured light |
| *Bettongia lesueur* | ANWC:Mammals:M12873 | Dry skeletonised | Structured light |
| *Bettongia penicillata* | SAMA:Mammalogy:M27628 | Dry skeletonised | Structured light |
| *Bettongia penicillata* | SAMA:Mammalogy:M27943 | Dry skeletonised | Structured light |
| *Bos taurus* | ANU:Zoo:M0198 | Dry skeletonised | Photogrammetry (cranium & partial postcrania), Structured light (partial postcrania) |
| *Bos taurus* | UQ:Zooarchaeology:208 | Dry skeletonised | Structured light |
| *Bos taurus* | UQ:Zooarchaeology:253 | Dry skeletonised | Structured light |
| *Bos taurus* | UQ:Zooarchaeology:271 | Dry skeletonised | Structured light |
| *Caloprymnus campestris* | MAGNT:Mammal:U8047 | Dry skeletonised | Structured light |
| *Caloprymnus campestris* | MAGNT:Mammal:U8048 | Dry skeletonised | μCT |
| *Caloprymnus campestris* | MAGNT:Mammal:U8051 | Dry skeletonised | Structured light |
| *Canis familiaris* | UQ:Zooarchaeology:165 | Dry skeletonised | Structured light |
| *Canis familiaris* | UQ:Zooarchaeology:52 | Dry skeletonised | Structured light |
| *Canis lupus dingo* | SAMA:Mammalogy:M5469 | Dry skeletonised | Structured light |
| *Caperea marginata* | SAMA:Mammalogy:M13636 | Wet | μCT |
| *Caperea marginata* | SAMA:Mammalogy:M6110 | Wet | μCT |
| *Capra hircus* | UQ:Zooarchaeology:183 | Dry skeletonised | Structured light |
| *Carettochelys insculpta* | MAGNT:Reptile:R35037 | Dry skeletonised | Structured light |
| *Casuarius bennetti* | FU:296 | Dry skeletonised | Structured light |
| *Casuarius casuarius* | ANWC:Birds:B21004 | Dry skeletonised | Structured light |
| *Casuarius casuarius* | ANWC:Birds:B49742 | Dry skeletonised | Structured light |
| *Casuarius casuarius* | SAMA:Ornithology:B46991 | Dry skeletonised | Structured light |
| *Casuarius unappendiculatus* | FU:299 | Dry skeletonised | Structured light |
| *Chaeropus ecaudatus* | SAMA:Mammalogy:M3971 | Wet (postcrania), Dry skeletonised (skull) | μCT |
| *Chaeropus yirratji* | SAMA:Mammalogy:M1618 | Dry skeletonised | μCT (skull & partial postcrania), Structured light (partial postcrania) |
| *Chelodina rugosa* | MAGNT:Reptile:R24813 | Dry skeletonised | Structured light |
| *Chelonia mydas* | MAGNT:reptile:R27163 | Dry skeletonised | Structured light |
| *Crocodylus novaeguineae* | MAGNT:Reptile:R16039 | Dry skeletonised | Structured light |
| *Crocodylus novaeguineae* | MAGNT:Reptile:R26444 | Dry skeletonised | Structured light |
| *Crocodylus porosus* | MAGNT:Reptile:R16036 | Dry skeletonised | Structured light |
| *Crocodylus porosus* | MAGNT:reptile:R38573 | Dry skeletonised | Structured light |
| *Dactylopsila megalura* | AM:Mammalogy:M.32374 | Dry skeletonised | Structured light |
| *Dactylopsila palpator* | AM:Mammalogy:M.37997 | Dry skeletonised | Structured light |
| *Dactylopsila trivirgata* | AM:Mammalogy:M.38910.003 | Dry skeletonised | Structured light |
| *Dactylopsila trivirgata* | AM:Mammalogy:M.9851 | Dry skeletonised | Structured light |
| *Dasycercus blythi* | SAMA:Mammalogy:M3635 | Wet | μCT |
| *Dasycercus cristicauda* | SAMA:Mammalogy:M25605 | Dry skeletonised | Structured light |
| *Dasycercus cristicauda* | SAMA:Mammalogy:M4573 | Dry skeletonised | Structured light |
| *Dasycercus cristicauda* | SAMA:Mammalogy:M27642 | Dry skeletonised | Structured light |
| *Dasyuroides byrnei* | SAMA:Mammalogy:M18284 | Dry skeletonised | Structured light |
| *Dasyurus albopunctatus* | AM:Mammalogy:M.13588 | Dry skeletonised | Structured light |
| *Dasyurus albopunctatus* | AM:Mammalogy:M.14854 | Dry skeletonised | Structured light |
| *Dasyurus geoffroyi* | AM:Mammalogy:PA.756 | Dry skeletonised | Structured light |
| *Dasyurus hallucatus* | SAMA:Mammalogy:M15386 | Wet | μCT |
| *Dasyurus hallucatus* | SAMA:Mammalogy:M7230 | Dry skeletonised | Structured light |
| *Dasyurus maculatus* | SAMA:Mammalogy:M27408 | Dry skeletonised | Structured light |
| *Dasyurus maculatus* | SAMA:Mammalogy:M6187 | Dry skeletonised | Structured light |
| *Dasyurus viverrinus* | SAMA:Mammalogy:M2086 | Dry skeletonised | Structured light |
| *Dasyurus viverrinus* | SAMA:Mammalogy:M7222 | Dry skeletonised | Structured light |
| *Delphinus delphis* | SAMA:Mammalogy:M18049 | Wet | μCT |
| *Delphinus delphis* | SAMA:Mammalogy:M25991 | Dry skeletonised | Structured light |
| *Dendrolagus bennettianus* | SAMA:Mammalogy:M1899 | Dry skeletonised | Structured light |
| *Dendrolagus bennettianus* | SAMA:Mammalogy:M5530 | Dry skeletonised | Structured light |
| *Dendrolagus dorianus* | AM:Mammalogy:M.32134 | Dry skeletonised | Structured light |
| *Dendrolagus dorianus* | AM:Mammalogy:M.9109 | Dry skeletonised | Structured light |
| *Dendrolagus goodfellowi* | AM:Mammalogy:M.23632.001 | Dry skeletonised | Structured light |
| *Dendrolagus goodfellowi* | AM:Mammalogy:M.7536 | Dry skeletonised | Structured light |
| *Dendrolagus goodfellowi* | AM:Mammalogy:M.9145 | Dry skeletonised | Structured light |
| *Dendrolagus inustus* | AM:Mammalogy:M.21840 | Dry skeletonised | Structured light |
| *Dendrolagus inustus* | AM:Mammalogy:M.25390 | Dry skeletonised | Structured light |
| *Dendrolagus lumholtzi* | FU:R402 | Dry skeletonised | Structured light |
| *Dendrolagus lumholtzi* | SAMA:Mammalogy:M1903 | Dry skeletonised | Structured light |
| *Dendrolagus lumholtzi* | SAMA:Mammalogy:M7206 | Dry skeletonised | Structured light |
| *Dendrolagus matschiei* | AM:Mammalogy:M6812 | Dry skeletonised | Structured light |
| *Dendrolagus mbaiso* | AM:Mammalogy:M.30719.002 | Dry skeletonised | Structured light |
| *Dendrolagus mbaiso* | AM:Mammalogy:M.30751.003 | Dry skeletonised | Structured light |
| *Dendrolagus mbaiso* | AM:Mammalogy:M.30754.001 | Dry skeletonised | Structured light |
| *Dendrolagus pulcherrinius* | AM:Mammalogy:M.21717 | Dry skeletonised | Structured light |
| *Dendrolagus spadix* | AM:Mammalogy:M.17212 | Dry skeletonised | Structured light |
| *Dendrolagus ursinus* | AM:Mammalogy:M.17230 | Dry skeletonised | Structured light |
| *Dendrolagus ursinus* | AM:Mammalogy:M.28159 | Dry skeletonised | Structured light |
| *Dobsonia magna* | SAMA:Mammalogy:M10475 | Wet | μCT |
| *Dorcopsis atrata* | AM:Mammalogy:M.19461 | Dry skeletonised | Structured light |
| *Dorcopsis hageni* | AM:Mammalogy:M.17202 | Dry skeletonised | Structured light |
| *Dorcopsis luctuosa* | SAMA:Mammalogy:M15178 | Dry skeletonised | Structured light |
| *Dorcopsis muelleri* | SAMA:Mammalogy:M12259 | Dry skeletonised | Structured light |
| *Dorcopsis muelleri* | SAMA:Mammalogy:M13754 | Dry skeletonised | Structured light |
| *Dorcopsulus vanheurni* | ANWC:Mammals:M15124 | Dry skeletonised | Structured light |
| *Dromaius novaehollandiae* | ANWC:Birds:B21011 | Dry skeletonised | Structured light |
| *Dromaius novaehollandiae* | SAMA:Ornithology:B31580 | Dry skeletonised |  |
| *Dugong dugon* | SAMA:Mammalogy:M847 | Dry skeletonised | Structured light |
| *Dugong dugon* | UQ:Zooarchaeology:257 | Dry skeletonised | Photogrammetry |
| *Echymipera kalubu* | MVZ:Mamm:MVZ:Mamm:138479 | Wet | μCT |
| *Echymipera rufescens* | SAMA:Mammalogy:M2820 | Wet | μCT |
| *Egernia cunninghami* | MCZ:Herp:R-130927 | Wet | μCT |
| *Elseya dentata* | MAGNT:Reptile:R24818 | Dry skeletonised | Structured light |
| *Elseya dentata* | MAGNT:Reptile:R27124 | Dry skeletonised | Structured light |
| *Equus caballus* | UQ:Zooarchaeology:201 | Dry skeletonised | Structured light |
| *Felis catus* | UQ:Zooarchaeology:176 | Dry skeletonised | Structured light |
| *Felis catus* | UQ:Zooarchaeology:38 | Dry skeletonised | Structured light |
| *Gallus gallus* | UQ:Zooarchaeology:200 | Dry skeletonised | Structured light |
| *Gymnobelideus leadbeateri* | SAMA:Mammalogy:M7377 | Dry skeletonised | Structured light |
| *Hemibelideus lemuroides* | MSU:MR:MR.4678 | Wet | μCT |
| *Hydromys chrysogaster* | SAMA:Mammalogy:M23213 | Wet | μCT |
| *Hyomys goliath* | AM:Mammalogy:M.15638.001 | Dry skeletonised | Structured light |
| *Hyperoodon planifrons* | SAMA:Mammalogy:M12796 | Dry skeletonised | Structured light |
| *Hypsiprymnodon moschatus* | SAMA:Mammalogy:M4154 | Wet | μCT |
| *Isoodon auratus* | SAMA:Mammalogy:M3078 | Wet | μCT |
| *Isoodon auratus* | SAMA:Mammalogy:M3995 | Wet | μCT |
| *Isoodon auratus* | SAMA:Mammalogy:M4734 | Wet | μCT |
| *Isoodon macrourus* | ANU:Zoo:AM79 | Dry skeletonised | Structured light |
| *Isoodon macrourus macrourus* | SAMA:Mammalogy:M7248 | Dry skeletonised | Structured light |
| *Isoodon obesulus obesulus* | SAMA:Mammalogy:M25975 | Wet | μCT |
| *Isoodon obesulus obesulus* | SAMA:Mammalogy:M7265 | Dry skeletonised | Structured light |
| *Lagenorhynchus cruciger* | SAMA:Mammalogy:M15715 | Wet | μCT |
| *Lagorchestes conspicillatus* | UQ:Zooarchaeology:278 | Dry skeletonised | Structured light |
| *Lagorchestes conspicillatus* | UQ:Zooarchaeology:392 | Dry skeletonised | Structured light |
| *Lagorchestes hirsutus* | MAGNT:Mammal:U6066 | Dry skeletonised | Structured light |
| *Lagorchestes hirsutus* | SAMA:Mammalogy:M3587 | Dry skeletonised | Structured light |
| *Lagostrophus fasciatus* | AM:Mammalogy:M.40035 | Dry skeletonised | Structured light |
| *Lagostrophus fasciatus fasciatus* | MAGNT:Mammal:U8082 | Dry skeletonised | Structured light |
| *Lasiorhinus krefftii* | AM:Mammalogy:M.16725.001 | Dry skeletonised | Structured light |
| *Lasiorhinus latifrons* | SAMA:Mammalogy:M23218 | Dry skeletonised | Structured light |
| *Leporillus apicalis* | SAMA:Mammalogy:M4074 | Wet | μCT |
| *Leporillus conditor* | SAMA:Mammalogy:M19635 | Wet | μCT |
| *Lepus europaeus* | L-EM:ES0033 | Wet | μCT |
| *Lissodelphis peronii* | SAMA:Mammalogy:M23161 | Wet | μCT |
| *Litoria caerulea* | UF:Herp:43434 | Wet | μCT |
| *Macropus fuliginosus* | SAMA:Mammalogy:M16578 | Dry skeletonised | Structured light |
| *Macropus giganteus* | SAMA:Mammalogy:M14523 | Dry skeletonised | Structured light |
| *Macropus giganteus giganteus* | MAGNT:Mammal:U8403 | Dry skeletonised | Structured light |
| *Macropus giganteus tasmaniensis* | MAGNT:Mammal:U8407 | Dry skeletonised | Structured light |
| *Macrotis lagotis* | SAMA:Mammalogy:M27631 | Dry skeletonised | Structured light |
| *Macrotis lagotis* | SAMA:Mammalogy:M3600 | Wet | μCT |
| *Macrotis lagotis* | SAMA:Mammalogy:M3602 | Dry skeletonised | μCT |
| *Macrotis leucura* | SAMA:Mammalogy:M3933 | Wet | μCT |
| *Mallomys aroaensis* | AM:Mammalogy:M.28073 | Dry skeletonised | Structured light |
| *Mallomys rothschildi* | AM:Mammalogy:M.13730.002 | Dry skeletonised | Structured light |
| *Mallomys rothschildi* | AM:Mammalogy:M.15626 | Dry skeletonised | Structured light |
| *Mesoplodon bowdoini* | SAMA:Mammalogy:M22559 | Dry skeletonised | Structured light |
| *Mesoplodon grayi* | SAMA:Mammalogy:M13975 | Wet | μCT |
| *Mesoplodon hectori* | SAMA:Mammalogy:M26434 | Dry skeletonised | Structured light |
| *Murexia longicaudata* | SAMA:Mammalogy:M2816 | Wet | μCT |
| *Myrmecobius fasciatus* | SAMA:Mammalogy:M3758 | Wet | μCT |
| *Notaden bennetti* | CAS:HERP:78115 | Wet | μCT |
| *Notamacropus agilis* | SAMA:Mammalogy:M23314 | Dry skeletonised | Structured light |
| *Notamacropus agilis* | SAMA:Mammalogy:M5979 | Dry skeletonised | Structured light |
| *Notamacropus dorsalis* | SAMA:Mammalogy:M2638 | Dry skeletonised | Structured light |
| *Notamacropus dorsalis* | SAMA:Mammalogy:M7967 | Dry skeletonised | Structured light |
| *Notamacropus eugenii* | ANU:Zoo:AM32 | Dry skeletonised | Structured light |
| *Notamacropus greyi* | SAMA:Mammalogy:M4988 | Dry skeletonised | Structured light |
| *Notamacropus irma* | SAMA:Mammalogy:M16489 | Dry skeletonised | Structured light |
| *Notamacropus parma* | SAMA:Mammalogy:M7191 | Dry skeletonised | Structured light |
| *Notamacropus parryi* | MAGNT:Mammal:U8323 | Dry skeletonised | Structured light |
| *Notamacropus parryi* | SAMA:Mammalogy:M14103 | Dry skeletonised | Structured light |
| *Notamacropus rufogriseus* | SAMA:Mammalogy:M16370 | Dry skeletonised | Structured light |
| *Notamacropus rufogriseus* | SAMA:Mammalogy:M7420 | Dry skeletonised | Structured light |
| *Notomys alexis* | SAMA:Mammalogy:M24165 | Wet | μCT |
| *Notomys mitchellii* | SAMA:Mammalogy:M24955 | Wet | μCT |
| *Notoryctes caurinus* | SAMA:Mammalogy:M3139 | Wet (postcrania), Dry skeletonised (skull) | μCT |
| *Notoryctes typhlops* | SAMA:Mammalogy:M4750 | Wet | μCT |
| *Onychogalea fraenata* | NTM:Mammal:U247 | Dry skeletonised | Structured light |
| *Onychogalea frenata* | SAMA:Mammalogy:M24347 | Dry skeletonised | Structured light |
| *Onychogalea lunata* | SAMA:Mammalogy:M1730 | Dry skeletonised | Structured light |
| *Onychogalea unguifera* | MAGNT:Mammal:U5329 | Dry skeletonised | Structured light |
| *Onychogalea unguifera* | SAMA:Mammalogy:M1728 | Dry skeletonised | Structured light |
| *Onychogalea unguifera* | SAMA:Mammalogy:M219 | Dry skeletonised | Structured light |
| *Orcaella heinsohni* | SAMA:Mammalogy:M26581 | Wet | μCT |
| *Ornithorhynchus anatinus* | ANU:Zoo:M438 | Dry skeletonised | Structured light |
| *Oryctolagus cuniculus* | L-EM:ES0162 | Wet | μCT |
| *Osphranter antilopinus* | MAGNT:Mammal:U6148 | Dry skeletonised | Structured light |
| *Osphranter antilopinus* | SAMA:Mammalogy:M8423 | Dry skeletonised | Structured light |
| *Osphranter bernardus* | MAGNT:Mammal:U6141 | Dry skeletonised | Structured light |
| *Osphranter robustus erubescens* | SAMA:Mammalogy:M13977 | Dry skeletonised | Structured light |
| *Osphranter robustus isabellinus* | UQ:Zooarchaeology:280 | Dry skeletonised | Structured light |
| *Osphranter rufus* | SAMA:Mammalogy:M16336 | Dry skeletonised | Structured light |
| *Ovis aries* | UQ:Zooarchaeology:276 | Dry skeletonised | Structured light |
| *Perameles bougainville* | SAMA:Mammalogy:M3657 | Wet | μCT |
| *Perameles bougainville* | SAMA:Mammalogy:M3973 | Wet | μCT |
| *Perameles bougainville* | SAMA:Mammalogy:M846 | Wet | μCT |
| *Perameles eremiana* | SAMA:Mammalogy:M3975 | Wet | μCT |
| *Perameles gunnii* | MAGNT:Mammal:U7600 | Dry skeletonised | Structured light |
| *Perameles nasuta* | MAGNT:Mammal:U7608 | Dry skeletonised | Structured light |
| *Peroryctes raffrayana* | AM:Mammalogy:M.30817 | Dry skeletonised | Structured light |
| *Petauroides volans* | SAMA:Mammalogy:M7326 | Dry skeletonised | Structured light |
| *Petauroides volans* | SAMA:Mammalogy:M8289 | Dry skeletonised | Structured light |
| *Petaurus australis* | MAGNT:Mammal:U7866 | Dry skeletonised | Structured light |
| *Petaurus breviceps* | MAGNT:Mammal:U0434 | Dry skeletonised | Structured light |
| *Petrogale brachyotis* | MAGNT:Mammal:U0103 | Dry skeletonised | Structured light |
| *Petrogale concinna* | MAGNT:Mammal:U3181 | Dry skeletonised | Structured light |
| *Petrogale concinna* | NTM:Mammal:U6117 | Dry skeletonised | Structured light |
| *Petrogale lateralis* | MAGNT:Mammal:U6060 | Dry skeletonised | Structured light |
| *Petrogale lateralis* | SAMA:Mammalogy:M24183 | Dry skeletonised | Structured light |
| *Petrogale lateralis lateralis* | SAMA:Mammalogy:M27400 | Dry skeletonised | Structured light |
| *Petrogale penicillata* | SAMA:Mammalogy:M27396 | Dry skeletonised | Structured light |
| *Petrogale penicillata* | SAMA:Mammalogy:M9539 | Dry skeletonised | Structured light |
| *Petrogale xanthopus* | SAMA:Mammalogy:M24737 | Dry skeletonised | Structured light |
| *Petrogale xanthopus* | SAMA:Mammalogy:M27390 | Dry skeletonised | Structured light |
| *Petropseudes dahli* | MAGNT:Mammal:U6099 | Dry skeletonised | Structured light |
| *Phalanger carmelitae* | SAMA:Mammalogy:M2901 | Wet | μCT |
| *Phalanger gymnotis* | AM:Mammalogy:M.21907 | Dry skeletonised | Structured light |
| *Phalanger gymnotis* | AM:Mammalogy:M.22157 | Dry skeletonised | Structured light |
| *Phalanger intercastellanus* | AM:Mammalogy:S.1892 | Dry skeletonised | Structured light |
| *Phalanger lullulae* | AM:Mammalogy:M.19788 | Dry skeletonised | Structured light |
| *Phalanger mimicus* | AM:Mammalogy:M.15759 | Dry skeletonised | Structured light |
| *Phalanger orientalis* | SAMA:Mammalogy:23273 | Wet | μCT |
| *Phalanger sericeus* | AM:Mammalogy:M.30752 | Dry skeletonised | Structured light |
| *Phalanger vestitus* | AM:Mammalogy:M.15791 | Dry skeletonised | Structured light |
| *Phascogale tapoatafa* | SAMA:Mammalogy:M3824 | Wet | μCT |
| *Phascolarctos cinereus* | FU:R404 | Dry skeletonised | Structured light |
| *Phocoena dioptrica* | SAMA:Mammalogy:M18940 | Wet | μCT |
| *Potorous longipes* | AM:Mammalogy:M.41268 | Dry skeletonised | Structured light |
| *Potorous platyops* | AM:Mammalogy:PA.1133.001 | Dry skeletonised | Structured light |
| *Potorous tridactylus* | SAMA:Mammalogy:M16233 | Dry skeletonised | Structured light |
| *Potorous tridactylus* | SAMA:Mammalogy:M7381 | Dry skeletonised | Structured light |
| *Pseudocheirus peregrinus* | SAMA:Mammalogy:M12547 | Wet | μCT |
| *Pseudochirops albertisii* | AM:Mammalogy:M.14589 | Dry skeletonised | Structured light |
| *Pseudochirops archeri* | AM:Mammalogy:M.148 | Dry skeletonised | Structured light |
| *Pseudochirops archeri* | AM:Mammalogy:M.8950 | Dry skeletonised | Structured light |
| *Pseudochirops cupreus* | AM:Mammalogy:M.16692.001 | Dry skeletonised | Structured light |
| *Pseudochirops cupreus* | AM:Mammalogy:M.30791.001 | Dry skeletonised | Structured light |
| *Pseudochirulus canescens* | AM:Mammalogy:S.1077 | Dry skeletonised | Structured light |
| *Pseudochirulus forbesi* | AM:Mammalogy:M.15691 | Dry skeletonised | Structured light |
| *Pseudochirulus herbertensis* | AM:Mammalogy:M.523 | Dry skeletonised | Structured light |
| *Pseudochirulus mayeri* | AM:Mammalogy:M.30679 | Dry skeletonised | Structured light |
| *Pseudochirulus mayeri* | AM:Mammalogy:M.9529 | Dry skeletonised | Structured light |
| *Pseudomys australis* | SAMA:Mammalogy:M17979 | Wet | μCT |
| *Pteropus neohibernicus* | AM:Mammalogy:M.28550.001 | Dry skeletonised | Structured light |
| *Pteropus poliocephalus* | AM:Mammalogy:M.20633 | Dry skeletonised | Structured light |
| *Pteropus poliocephalus* | AM:Mammalogy:M.42886.003 | Dry skeletonised | Structured light |
| *Rhinella marina* | UF:Herp:172560 | Wet | μCT |
| *Sarcophilus harrisi* | MAGNT:Mammal:U7559 | Dry skeletonised | Structured light |
| *Sarcophilus harrisii* | SAMA:Mammalogy:M7198 | Dry skeletonised | Structured light |
| *Setonix brachyurus* | SAMA:Mammalogy:M14102 | Dry skeletonised | Structured light |
| *Sminthopsis crassicaudata* | SAMA:Mammalogy:M12809 | Wet | μCT |
| *Sousa sahulensis* | SAMA:Mammalogy:M26583 | Wet | μCT |
| *Spilocuscus maculatus* | AM:Mammalogy:M.11634 | Dry skeletonised | Structured light |
| *Sus scrofa* | UQ:Zooarchaeology:168 | Dry skeletonised | Structured light |
| *Sus scrofa* | UQ:Zooarchaeology:195 | Dry skeletonised | Structured light |
| *Sus scrofa* | UQ:Zooarchaeology:260 | Dry skeletonised | Structured light |
| *Tachyglossus aculeatus* | ANU:Zoo:AM504 | Dry skeletonised | Structured light |
| *Tasmacetus shepherdi* | SAMA:Mammalogy:M12774 | Dry skeletonised | Structured light |
| *Threskiornis molucca moluccus* | FU:R:574 | Dry skeletonised | μCT |
| *Thylacinus cynocephalus* | SAMA:Mammalogy:M1960 | Dry skeletonised | Structured light |
| *Thylacinus cynocephalus* | SAMA:Mammalogy:M95 | Dry skeletonised | Structured light |
| *Thylogale billardierii* | FU:R213 | Dry skeletonised | Structured light |
| *Thylogale billardierii* | MAGNT:Mammal:U8190 | Dry skeletonised | Structured light |
| *Thylogale billardierii* | SAMA:Mammalogy:M2868 | Dry skeletonised | Structured light |
| *Thylogale browni* | AM:Mammalogy:M.32133 | Dry skeletonised | Structured light |
| *Thylogale brunii* | AM:Mammalogy:M.20214 | Dry skeletonised | Structured light |
| *Thylogale calabyi* | AM:Mammalogy:M.38070 | Dry skeletonised | Structured light |
| *Thylogale calabyi* | AM:Mammalogy:M.38073 | Dry skeletonised | Structured light |
| *Thylogale stigmatica* | AM:Mammalogy:M.54091 | Dry skeletonised | Structured light |
| *Thylogale stigmatica* | AM:Mammalogy:M38878 | Dry skeletonised | Structured light |
| *Thylogale stigmatica* | MAGNT:Mammal:U8203 | Dry skeletonised | Structured light |
| *Thylogale thetis* | ANU:Zoo:AM169 | Dry skeletonised | Structured light |
| *Tiliqua scincoides* | CAS:HERP:254658 | Wet | μCT |
| *Trichosurus arnhemensis* | MAGNT:Mammal:U5993 | Dry skeletonised | Structured light |
| *Trichosurus caninus* | MAGNT:Mammal:U7946 | Dry skeletonised | Structured light |
| *Trichosurus vulpecula* | SAMA:Mammalogy:M20643 | Dry skeletonised | Structured light |
| *Trichosurus vulpecula* | UQ:Zooarchaeology:102 | Dry skeletonised | Structured light |
| *Tursiops aduncus* | SAMA:Mammalogy:M15598 | Wet | μCT |
| *Tursiops aduncus* | SAMA:Mammalogy:M24893 | Dry skeletonised | Structured light |
| *Uromys anak* | AM:Mammalogy:M.24234.001 | Dry skeletonised | Structured light |
| *Uromys caudimaculatus* | SAMA:Mammalogy:M14794 | Wet | μCT |
| *Uromys neobritannicus* | AM:Mammalogy:M.20689 | Dry skeletonised | Structured light |
| *Uromys rex* | AM:Mammalogy:M.13594 | Dry skeletonised | Structured light |
| *Vombatus ursinus* | SAMA:Mammalogy:M26232 | Dry skeletonised | Structured light |
| *Vombatus ursinus tasmaniensis* | SAMA:Mammalogy:M11977 | Dry skeletonised | Structured light |
| *Vulpes vulpes* | ANU:Zoo:M0002 | Dry skeletonised | Structured light |
| *Wallabia bicolor* | SAMA:Mammalogy:M11364 | Dry skeletonised | Structured light |
| *Wyulda squamicaudata* | MAGNT:Mammal:U7890 | Dry skeletonised | Structured light |
| *Xenuromys barbatus* | AM:Mammalogy:M.13767.002 | Dry skeletonised | Structured light |
| *Xenuromys barbatus* | AM:Mammalogy:M.15675 | Dry skeletonised | Structured light |
| *Zaglossus attenboroughi* | AM:Mammalogy:M.9852.002 | Dry skeletonised | Structured light |
| *Zaglossus bartoni* | AM:Mammalogy:M.8263.002 | Dry skeletonised | Structured light |
| *Zaglossus bruijni* | AM:Mammalogy:M.34012 | Dry skeletonised | Structured light |
